# Growth Differentiation Factor 9 Supplementation Restores the Lipopolysaccharide‐Induced Negative Effects on Bovine Oocyte Developmental Competence

**DOI:** 10.1002/mrd.70099

**Published:** 2026-04-09

**Authors:** Dragos Scarlet, Idil Serbetci, Gerhard Schuler, Heinrich Bollwein, Mariusz P. Kowalewski

**Affiliations:** ^1^ Institute of Veterinary Anatomy, Vetsuisse Faculty Zurich Zurich Switzerland; ^2^ Clinic of Reproductive Medicine, Vetsuisse Faculty Zurich Zurich Switzerland; ^3^ Veterinary Clinic for Reproductive Medicine and Neonatology Justus‐Liebig‐University Giessen Germany; ^4^ AgroVet‐Strickhof, Vetsuisse Faculty Lindau Switzerland

**Keywords:** bovine, cumulus cells, GDF9, LPS, oocyte

## Abstract

Growth differentiation factor 9 (GDF9) plays a key role in enhancing developmental competence and blastocyst yield during in vitro maturation (IVM). This study investigated whether GDF9 could counteract the harmful effects of lipopolysaccharide (LPS), a gram‐negative bacterial component, on bovine in vitro embryo production, while also examining related gene expression in cumulus cells and oocytes. We hypothesized that GDF9 may compensate for LPS through the NF‐κB pathway. Ovaries were collected from a slaughterhouse, and oocytes (≥ 50 per group per replicate) were matured under four conditions: control, GDF9, LPS, and GDF9 + LPS. After IVM, fertilization and culture were performed, with blastocyst development evaluated on Day 7. Cumulus cells and oocytes were analyzed for gene expression (RT‐qPCR), while IVM media were tested for progesterone and estradiol. Results showed that co‐treatment with GDF9 and LPS restored cumulus expansion, cleavage, blastocyst rate, and embryo quality compared with LPS alone (*p* < 0.05), with no differences from controls. LPS increased mRNA levels of *CXCL8*, *TNFα*, and *TLR2* in cumulus cells (*p* < 0.05), but candidate gene expression in oocytes and cumulus cells remained unaffected. Steroid concentrations and estradiol:progesterone ratios were similar across groups. In summary, GDF9 supplementation alleviates LPS‐induced impairment in bovine oocytes during IVM, though the underlying mechanisms remain unclear.

## 1 Introduction

Acquisition of oocyte developmental competence depends on a healthy intrafollicular environment, with active communication between oocytes and granulosa cells regulating oocyte growth and maturation. This bidirectional communication is essential for supplying the oocyte with essential molecules for proper metabolism and regulation of meiotic maturation (Y. Q. Su et al. 2008). In this regard, the oocyte secretes growth differentiation factor 9 (GDF9) and bone morphogenetic protein 15 (BMP15), which play an essential role in follicular development and control proliferation, development and expansion of granulosa cells in farm animals such as sheep, cattle, and horses (Davis et al. 2018; Juengel et al. 2009, 2004; Samie et al. 2025). These two factors act either as biologically active heterodimers or as homodimers in a synergistic cooperation (Sanfins et al. 2018). Their interaction induces cholesterol biosynthesis in mouse cumulus cells (Y. Q. Su et al. 2008), whereas GDF9 alone promotes cell proliferation and reduces steroid hormone production by the granulosa cells in cattle (Spicer et al. 2006), thereby preventing premature differentiation and supporting follicular integrity and antrum formation (Alam et al. 2018).

In the ovary, GDF9 is generally secreted by the oocyte from the primordial or primary follicle stage onwards, depending on the species, but it is also expressed by the cumulus cells in bovine (Hosoe et al. 2011; Samie et al. 2025; Sun et al. 2010). During in vitro maturation (IVM), the levels of GDF9 mRNA significantly decrease in the oocytes of pigs and horses, while, in pigs, the protein expression was shown to remain unchanged, suggesting posttranscriptional regulation (Lin et al. 2014; Scarlet et al. 2017). In humans, transition to metaphase II does not influence GDF9 protein expression in oocytes but is associated with reduced levels of GDF9 in the IVM spent medium (Cadenas et al. 2022). Furthermore, the addition of GDF9 to IVM media increases the developmental competence of bovine oocytes and the number of blastocysts produced (Hussein et al. 2011; J. Su et al. 2014), most likely by influencing cumulus cell metabolism (Eppig et al. 2005), whereas pharmacological inhibition of GDF9 signaling in porcine oocytes impairs meiotic maturation, embryo development, and maternal gene expression (Lin et al. 2014). Together, these findings support the functionality of the GDF9 signaling system in bovine granulosa cells and its involvement in follicular selection and coordinated follicular development (Jayawardana et al. 2006).

As a member of the transforming growth factor‐beta (TGF‐β) superfamily, GDF9 primarily exerts its action through its specific type I receptor activin receptor‐like kinase 5 (ALK5), followed by the activation of the *Sma* and mothers against decapentaplegic (SMAD)2/3 pathway (Mazerbourg et al. 2004). Besides the canonical pathway, studies in rats have shown that granulosa cells also respond to the synergistic treatment with GDF9 and BMP15 by activating the NF‐κB noncanonical pathway, among others (Reader et al. 2016).

The reciprocal signaling between the oocyte and granulosa cells implies that pathological conditions affecting follicular development can adversely influence oocyte quality during the acquisition of developmental competence. Uterine and/or mammary gland inflammation have been shown to decrease reproductive performance of dairy cows and also cause long‐term reproductive dysfunction although the mechanisms for this have not yet been fully elucidated (LeBlanc 2008). One of the most pathogenic bacteria affecting the reproductive performance of dairy cattle is *Escherichia coli* (*E. coli*; Goulart and Mellata 2022; Williams et al. 2007), whose effects are primarily mediated by lipopolysaccharide (LPS). In diseased cows, LPS is detected not only in plasma, but also in follicular fluid, uterine fluid, and milk (Hakogi et al. 1989; Herath et al. 2007; Mateus et al. 2003), therefore exerting local effects on normal organ function. The presence of LPS in the follicular fluid after natural infection reduces estradiol‐17β (E2) and increases progesterone (P4) concentration by altering the activity of steroidogenic enzymes (Magata et al. 2014). Similar observations were made in vitro, as the steroidogenic and proliferative capacity of bovine granulosa cells, as well as their E2 production, were suppressed in response to LPS challenge (Magata et al. 2014; Shimizu et al. 2012; Yamamoto et al. 2023).

Bacterial LPS effects on bovine granulosa cells are initiated through toll‐like receptor 2 (TLR2) and 4 (TLR4) (Price et al. 2013). After binding to the receptors, LPS activates MAPK and nuclear factor‐κB (NF‐κB) pathways, resulting in an increased abundance of inflammatory cytokines such as interleukin (IL)1β, IL6, IL8, and tumor necrosis factor alpha (TNFα) (Price et al. 2013). Interestingly, TLR2 and TLR4 are also present on bovine oocytes and cumulus cells (Magata and Shimizu 2017). Moreover, LPS impairs meiosis and mitochondrial function of oocytes, as well as their subsequent capacity to develop to the blastocyst stage (Castro et al. 2024; Magata et al. 2024; Magata and Shimizu 2017). This effect seems to be mediated by granulosa cells and their products in response to LPS rather by direct effects on the oocyte itself (Tariq et al. 2025). Consistent with this, LPS exposure reduces transzonal projection density in porcine cumulus–oocyte complexes (COCs) and disrupts gap junctional communication in ovine reproductive tissues (M. Chen et al. 2025; Gram et al. 2019). Importantly, decreased GDF9 levels have been reported in follicular fluid from bovine ovarian cysts and in preantral follicles of cows with chronic mastitis, coinciding with elevated LPS concentrations (Çolakoğlu et al. 2020; Rahman et al. 2012). Together, these findings suggest that inflammatory stress, mediated by LPS, compromises follicular development at least in part by disrupting oocyte‐derived GDF9 signaling.

Considering the direct effects of both GDF9 and LPS on granulosa cell function and oocyte developmental competence, as well as their possible interactions, this study aimed to analyze whether GDF9 could alleviate LPS‐induced negative effects during bovine in vitro embryo production, as well as changes in gene expression in cumulus cells and oocytes associated with this interaction. We hypothesized that GDF9 addition to the oocyte IVM media would stimulate cumulus expansion, reduce apoptosis, and stabilize an adequate steroidogenic capacity of granulosa cells, thereby securing the development of blastocysts derived from LPS‐challenged oocytes.

## 2 Material and Methods

### 2.1 Ovary Collection and Oocyte Selection

**Figure 1:**
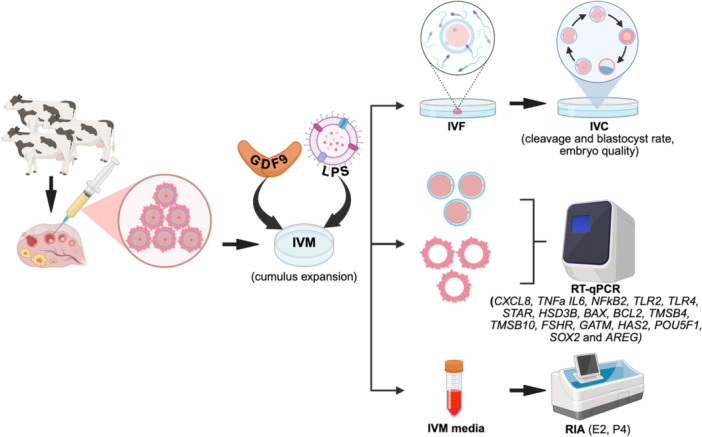
Schematic illustration of the experimental design. Cumulus–oocyte complexes were collected and subjected to IVM for 20–22 h either in control medium alone, or in medium containing 150 ng/mL recombinant human GDF9, in the presence of 0.1 µg/mL *E. coli* LPS, or both 0.1 µg/mL LPS and 150 ng/mL recombinant human GDF9 simultaneously. Afterwards, IVF and IVC were performed for a total of seven independent experiments. From a different set of four independent experiments, cumulus cells and oocytes were separated and collected for gene expression analysis, while the IVM media were collected for hormone assays in the spent culture media. Analyzed parameters in each step are given in brackets. Created in BioRender. Scarlet, D. (2026) https://BioRender.com/sswunkv.

Bovine ovaries obtained from a local slaughterhouse were transported to the laboratory in prewarmed 0.9% physiological saline solution at 37°C (B. Braun Medical AG, Sempach, Switzerland) within 3 h of slaughter. Upon arrival, ovaries were washed at least three times with prewarmed saline solution at 37°C to remove excess blood and debris. The COCs were aspirated from follicles (2–8 mm in diameter) with a 18G hypodermic needle attached to a syringe and collected into 50 mL tubes (Corning, USA) with BoviFlush (Minitube, Tiefenbach, Germany) and held at 38°C. At the end of collection, the COCs were classified under a stereomicroscope and only those containing more than three layers of compact cumulus cells and homogeneous cytoplasm (Grades 1 and 2 according to IETS criteria: https://www.iets.org/Publications/IETS‐Manual) were selected for IVM. A schematic illustration of the experimental design used in this study is presented in Figure 1.

### 2.2 In Vitro maturation (IVM)

Selected COCs were immediately transferred to BO‐WASH (IVF Bioscience, Cornwall, United Kingdom) and washed two additional times. IVM was carried out in groups of 10 COCs in 100 μL of BO‐IVM (IVF Bioscience) under mineral oil (Sigma‐Aldrich, GmbH, Buchs, Switzerland) at 38.5°C with 5% CO_2_ and saturated humidity for 20–22 h. The experimental conditions included a control group using only BO‐IVM, a group treated with 0.1 µg/mL *E. coli* LPS (O55:B5; Sigma‐Aldrich GmbH) in BO‐IVM, a group treated with 150 ng/mL recombinant human GDF9 (SRP4872, Sigma‐Aldrich GmbH) in BO‐IVM, and a group treated with both 0.1 µg/mL LPS and 150 ng/mL recombinant human GDF9 in BO‐IVM. The LPS concentration used in this study was previously shown to exert adverse effects on blastocyst development in this species (Castro et al. 2024; Magata and Shimizu 2017) and is similar to the mean intrafollicular concentration determined in cows with clinical endometritis (Herath et al. 2007; Yamamoto et al. 2023). In case of GDF9, the concentration used lies within the biologically active window (100–200 ng/mL) as previously shown (Alam et al. 2018; McNatty et al. 2005; J. Su et al. 2014), while representing an intermediate, physiology‑oriented dose that allows assessment of potential nonlinear dose–response effects.

In each experiment, at least 50 COCs were included per treatment. After maturation, each COC was examined individually under a stereomicroscope by a single evaluator who was blinded to the experimental groups. COCs were considered expanded if they showed complete expansion, including the innermost corona radiata cells. The degree of cumulus expansion was then expressed as the percentage of expanded COCs out of the total number of COCs in each group for every experiment. Additionally, the IVM medium from each group was collected in 1.5 mL Eppendorf PCR tubes and centrifuged at 1000*g* for 10 min. The supernatant was collected and stored at −20°C for steroid analyses. IVM was performed in the same way for in vitro embryo production as well as for molecular and steroid hormone analyses, according to the standard laboratory protocol.

### 2.3 Total RNA Extraction, Reverse Transcription, and Semi‐Quantitative Real‐Time TaqMan qPCR

After 20–22 h of IVM, COCs from five independent experiments were transferred to 80 IU/mL hyaluronidase (GM501, Gynemed, Sierksdorf, Germany) for 5 min at 37°C to remove the oocytes from the cumulus cells. After denudation, the oocytes were isolated and washed in BO‐IVM before being transferred to 1.5 mL Eppendorf PCR tubes. The remaining fluid containing the cumulus cells was collected, diluted in 1 mL sterile PBS, and centrifuged at 1000*g*, at 4°C for 10 min. After centrifugation, the supernatant was carefully discarded and a second washing step performed to remove residual hyaluronidase. Both oocytes and cumulus cells were then stored at −80°C for further analysis.

The expression of selected target genes from different functional groups, including inflammation markers, LPS signaling pathway, markers of cumulus expansion, indicators of meiotic resumption and oocyte developmental competence, steroidogenic markers, apoptosis markers, as well as pluripotency markers, was determined in the collected cumulus cells and oocytes. Total RNA was extracted from the cumulus cells and oocytes using TRIzol reagent, according to the manufacturer's guidelines (Invitrogen, Carlsbad, CA, USA), and RNA concentration and purity were assessed with a NanoDrop 2000 Spectrophotometer (Thermo Fisher, Foster City, CA, USA). A total of 1.3 μg of RNA per sample was treated with DNase using the RQ1 RNase‐free DNase kit (Promega, Duebendorf, Switzerland). Complementary DNA (cDNA) synthesis was performed with the MultiScribe Reverse Transcriptase and random hexamers (Applied Biosystems by Thermo Fisher). Semi‐quantitative real‐time PCR (TaqMan) was carried out in duplicate in 96‐well optical plates, employing cDNA corresponding to 100 ng of RNA per sample and the FastStart Universal Probe Master (ROX, Roche Diagnostics AG, Basel, Switzerland). Autoclaved water was used as a negative control, and a minus RT control was routinely included to confirm the absence of genomic DNA contamination. The TaqMan systems used are detailed in Table 1. Target gene expression was quantified by the comparative Ct method (ΔΔCt) and normalized to the geometric mean of the reference genes *ACTB* and *GAPDH*, which are commonly used for gene expression analyses of bovine oocytes and cumulus cells (M. Chen et al. 2025; Fu et al. 2023; Magata et al. 2024; Piersanti et al. 2019; Turhan et al. 2020).

**Table 1:** List of symbols, corresponding gene names, and TaqMan systems used for semi‐quantitative real‐time TaqMan PCR.

Gene symbol	Gene name	GenBank access number	Product number/reference
*IL6*	Interleukin 6	NM_173923.2	Bt03211905_m1
*CXCL8*	Interleukin 8	NM_001009401.2	Bt03211906_m1
*TNFα*	Tumor necrosis factor alpha	NM_173966.3	Bt03259154_m1
*NF‐kB*	Nuclear factor kappa B subunit 2	NM_001102101.1	Bt03272779_m1
*TLR2*	Toll‐like receptor 2	NM_174197.2	Lüttgenau et al. (2016)
*TLR4*	Toll‐like receptor 4	NM_174198.6	Lüttgenau et al. (2016)
*STAR*	Steroidogenic acute regulatory protein	NM_174189	Turhan et al. (2020)
*HSD3B*	3beta‐hydroxysteroid dehydrogenase	NM_174343	Turhan et al. (2020)
*BAX*	BCL2‐associated X, apoptosis regulator	NM_173894.1	Bt01016551_g1
*BCL2*	BCL2 apoptosis regulator	NM_001166486.1	Bt04298952_m1
*TMSB4*	Thymosin beta‐4	NM_001113231.3	Turhan et al. (2020)
*TMSB10*	Thymosin beta‐10	NM_174623.2	Turhan et al. (2020)
*FSHR*	Follicle‐stimulating hormone receptor	NM_174061.1	Bt03212674_m1
*GATM*	Glycine amidinotransferase	NM_001045878.1	Bt03237895_m1
*HAS2*	Hyaluronan synthase 2	NM_174079.3	Bt03212695_g1
*AREG*	Amphiregulin	NM_001099092.1	Bt03271012_m1
*POU5F1*	POU class 5 homeobox 1	NM_174580.3	Bt03223846_g1
*SOX2*	SRY‐box transcription factor 2	NM_001105463.2	Bt03278318_s1
*ACTB*	Actin beta	NM_173979.3	Bt03279175_g1
*GAPDH*	Glyceraldehyde‐3‐ phosphate dehydrogenase	NM_001034034	Turhan et al. (2020)

### 2.4 Hormone Assays

The IVM medium (control, GDF9, LPS, and GDF9 + LPS) collected at the end of each IVM round from COCs undergoing IVF/IVC was analyzed for progesterone (P4) and estradiol‐17β (E2) content by in‐house competitive radioimmunoassays (RIAs) as previously described (Scarlet et al. 2025). The assays were previously validated for bovine species (Hoffmann 1977). The sample volume was 50 µL for the P4 measurement and 100 µL for the E2 measurement. All samples (*n* = 28, 7/treatment) were assayed in duplicate. The cross‐reactivity of the P4 antiserum with other steroids was: androstenedione < 0.01%, DHEA < 0.01%, estradiol < 0.01%, estrone < 0.01%, cortisol < 0.01%, testosterone 0.37%, 17α‐hydroxy‐pregnenolone < 0.01%, 17α‐hydroxy‐progesterone 0.49%, and pregnenolone 0.69%. The cross‐reactivity of the E2 antiserum with other steroids was: estrone 1.30%, estriol 0.68%, androstenedione < 0.01%, cortisol < 0.01%, DHEA < 0.01%, pregnenolone < 0.01%, P4 < 0.01%, testosterone < 0.01%, and 5a‐dihydro‐testosterone < 0.01%. For P4, the lower limit of detection was 0.2 ng/mL, and the intra‐ and interassay coefficients of variation were 8.8% and 8.9%, respectively. For E2, the lower limit of detection was 5 pg/mL, and the intra‐ and interassay coefficients of variation were 7.1% and 12.0%, respectively.

### 2.5 In Vitro Fertilization (IVF) and Culture (IVC)

In vitro embryo production was performed in seven independent experiments as previously described (Serbetci et al. 2024), with slight modifications. After thawing in a water bath at 37.2°C for 30 s, semen was place on top of 1 mL 80% BoviPure (Nidacon, Mölndal, Sweden) and centrifuged for 6 min at 3700*g*. After removing the supernatant, the pellet was resuspended in 1 mL of BO‐SEMEN PREP (IVF Bioscience) and centrifuged for 2 min at 3700*g*. Following selection, the sperm concentration was determined using computer‐assisted sperm analysis. For IVF, 1 × 10^6^ spermatozoa/mL were co‐incubated with up to 15 COCs in 50 μL droplets of BO‐IVF (IVF Bioscience) overlaid with mineral oil (Sigma‐Aldrich GmbH) at 38.5°C, under an atmosphere of 5% CO_2_ in air and saturated humidity. All IVF runs were performed with frozen semen from the same ejaculate of one Simmental bull with known in vitro fertility. The day of IVF was defined as Day 0. Twenty hours post‐IVF, surrounding cumulus cells were removed from the presumptive zygotes using a 135 μm stripper pipette (CooperSurgical, Inc., Trumbull, CT, United States). Afterwards, groups of 10 presumptive zygotes were placed in 40 μL droplets of BO‐IVC (IVF Bioscience) covered with mineral oil (Sigma‐Aldrich) and cultured at 38.5°C, under an atmosphere of 5% CO_2_, 5% O_2_, and saturated humidity. Two days (48 h) after IVF, the cleavage rate was evaluated, and the noncleaved zygotes were removed from the culture drops and discarded. The cleaved embryos were cultured until Day (d) 7, with the blastocyst rates evaluated on d6 and d7. Only expanded (Stage Code 7), hatching (Stage Code 8), and hatched (Stage Code 9) blastocysts were considered. The quality of the embryos was assessed as excellent (Code 1), fair (Code 2), poor (Code 3), or degenerated (Code 4), according to the recommendations of the 5th IETS Manual (https://www.iets.org/Publications/IETS‐Manual).

### 2.6 Statistical Analysis

The computer software GraphPad Prism version 10 (Boston, MA, USA) was used for all analyses. To evaluate differences in IVP outcome, cumulus cell and oocyte gene expression, as well as steroid hormone concentrations in the IVM medium from the different treatment groups, a two‐way ANOVA was applied and, if the *p* value was < 0.05, a Tukey multiple comparisons post hoc test was performed. All PCR results are presented as geometric mean ± geometric standard deviation, while hormone concentrations are presented as mean ± SD.

## 3 Results

### 3.1 GDF9 Restores the LPS‐Induced Impairment in Bovine Oocyte Development During In Vitro Embryo Production

**Figure 2:**
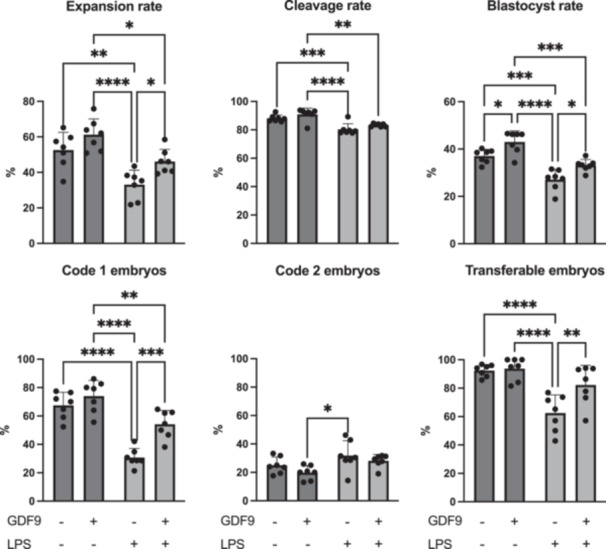
Effect of growth differentiation factor 9 (GDF9) during IVM on developmental potential of lipopolysaccharide (LPS) challenged bovine oocytes (*n* = 7 independent experiments). Cumulus–oocyte complexes were cultured for 20–22 h either in control medium alone, or in medium containing 150 ng/mL recombinant human GDF9, in the presence of 0.1 µg/mL *E. coli* LPS, or both 0.1 µg/mL LPS and 150 ng/mL recombinant human GDF9 simultaneously. A two‐way ANOVA was applied to test for treatment effects and, if the *p* value was < 0.05, a Tukey multiple comparison post hoc test was performed. Different superscripts indicate significant differences (*p < 0.05, **p < 0.01, ***p < 0.001, ****p < 0.0001).

Cumulus cell expansion was significantly reduced after LPS treatment (33.1% ± 8.1%) compared to all other groups (control: 52.6% ± 10.0%, GDF9: 61.3% ± 8.9%, GDF9/LPS: 46.1% ± 6.8%, *p* < 0.05 in all cases, Figure 2). Cleavage rates were higher in control (88.0% ± 2.3%) and GDF9 (90.8% ± 4.4%) compared to LPS (80.3% ± 4.0%) (*p* < 0.05 in both cases), but were similar between control and GDF9/LPS (83.2% ± 1.0%) (Figure 2). Blastocyst rates (control: 37.0% ± 2.8%, GDF9: 43.0% ± 4.7%, LPS: 27.1% ± 4.4%, GDF9/LPS: 33.1% ± 2.5%) were increased following GDF9 treatment (*p* < 0.05 in all cases), and decreased following LPS treatment (*p* < 0.05 in all cases) (Figure 2). The addition of GDF9 to LPS yielded similar results to the control. Furthermore, blastocyst formation was also reflected in the percentage of Code 1 embryos. Specifically, there were fewer Code 1 blastocysts following LPS treatment (30.7% ± 6.4%) compared to all other groups (control: 67.5% ± 9.2%, GDF9: 73.9% ± 11.1%, GDF9/LPS: 54.1% ± 9.8%, *p* < 0.05 in all cases, Figure 2), whereas results did not differ between control and GDF9/LPS groups (*p* > 0.05). Also, when the percentage of transferable embryos (Codes 1 and 2) was compared, results did not differ between control (92.4% ± 4.2%), GDF9 (93.7% ± 7.8%), and GDF9/LPS (82.2% ± 13.8%), but were significantly lower (*p* < 0.05 in all cases) in the LPS group (62.5% ± 12.8%) (Figure 2).

### 3.2 GDF9 Does Not Influence the Expression of Selected Target Genes in Bovine COCs

**Figure 3:**
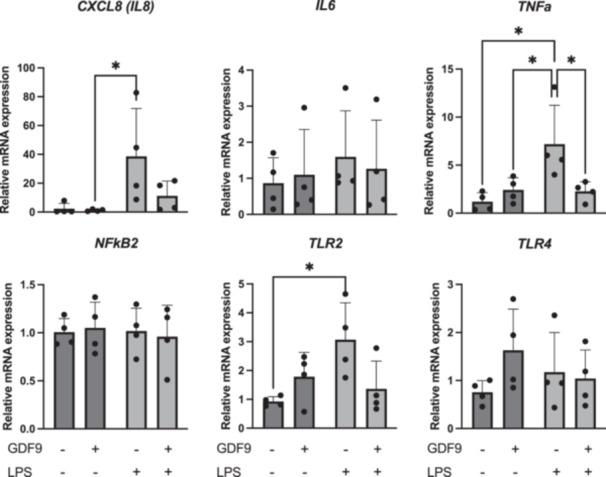
Effect of growth differentiation factor 9 (GDF9) on cytokine or LPS signaling pathway‐related gene expression in bovine cumulus cells after lipopolysaccharide (LPS) challenge during IVM (*n* = 4 independent experiments). Cumulus–oocyte complexes were cultured for 20–22 h either in control medium alone, or in medium containing 150 ng/mL recombinant human GDF9, in the presence of 0.1 µg/mL *E. coli* LPS, or both 0.1 µg/mL LPS and 150 ng/mL recombinant human GDF9 simultaneously. Cumulus cells were subjected to gene expression analysis. A two‐way ANOVA was applied to test for treatment effects and, if the *p* value was < 0.05, a Tukey multiple comparison post hoc test was performed. Relative gene expression is presented as determined by semi‐quantitative real‐time (TaqMan) PCR (Xg ± SD). Different superscripts indicate significant differences (*p < 0.05).

**Figure 4:**
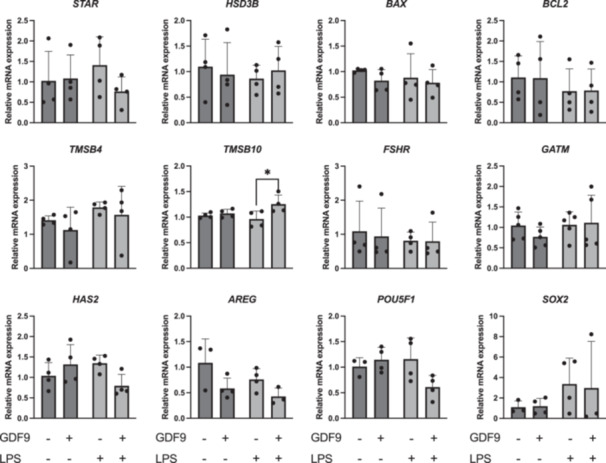
Effect of growth differentiation factor 9 (GDF9) on steroidogenesis, apoptosis, cumulus expansion, and the acquisition of developmental competence or pluripotency‐related gene expression in bovine cumulus cells after lipopolysaccharide (LPS) challenge during IVM (*n* = 4 independent experiments). Cumulus–oocyte complexes were cultured for 20–22 h either in control medium alone, or in medium containing 150 ng/mL recombinant human GDF9, in the presence of 0.1 µg/mL *E. coli* LPS, or both 0.1 µg/mL LPS and 150 ng/mL recombinant human GDF9 simultaneously. Cumulus cells were subjected to gene expression analysis. A two‐way ANOVA was applied to test for treatment effects and, if the *p* value was < 0.05, a Tukey multiple comparison post hoc test was performed. Relative gene expression is presented as determined by semi‐quantitative real‐time (TaqMan) PCR (Xg ± SD). Different superscripts indicate significant differences (*p < 0.05).

**Figure 5:**
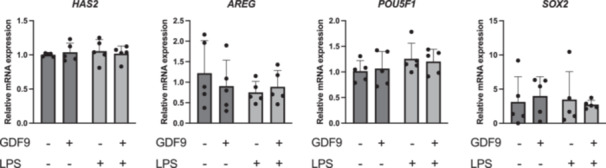
Effect of growth differentiation factor 9 (GDF9) on bovine oocyte gene expression after lipopolysaccharide (LPS) challenge during IVM (*n* = 5 independent experiments). Cumulus–oocyte complexes were cultured for 20–22 h either in control medium alone, or in medium containing 150 ng/mL recombinant human GDF9, in the presence of 0.1 µg/mL *E. coli* LPS, or both 0.1 µg/mL LPS and 150 ng/mL recombinant human GDF9 simultaneously. Oocytes were subjected to gene expression analysis. A two‐way ANOVA was applied to test for treatment effects and, if the *p* value was < 0.05, a Tukey multiple comparison post hoc test was performed. Relative gene expression is presented as determined by semi‐quantitative real‐time (TaqMan) PCR (Xg ± SD).

An increased mRNA abundance of *CXCL8* was observed in cumulus cells in response to LPS challenge compared to control (*p* = 0.05) and GDF9 (*p* < 0.05) (Figure 3). Also, an increased mRNA abundance of TNFa was observed following LPS treatment compared to all other treatments (*p* < 0.05 in all cases), whereas no difference was seen between GDF9/LPS and control (*p* > 0.05) (Figure 3). No changes were noticed in the mRNA expression of genes involved in the LPS signaling pathway such as *NFkB2* and *TLR4* (*p* > 0.05 in both cases), but there was a significant increase in *TLR2* levels following LPS treatment compared to control (*p* < 0.05) (Figure 3). The mRNA abundance of genes involved in steroidogenesis (*STAR*, *HSD3B*), apoptosis (*BAX*, *BCL2*), cumulus expansion and acquisition of developmental competence (*TMSB4*, *FSHR*, *GATM*, *HAS2*, *AREG*), or pluripotency (*POU5F1*, *SOX2*) in cumulus cells was not affected by treatment (Figure 4). Similarly, the transcriptional levels of *HAS2*, *AREG*, *POU5F1*, and *SOX2* in oocytes were not affected by the treatments (Figure 5).

### 3.3 GDF9 Does Not Affect Steroid Hormone Production by Bovine COCs During IVM

**Figure 6:**
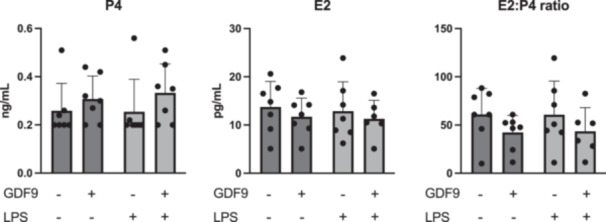
Effect of growth differentiation factor 9 (GDF9) on bovine cumulus cells steroid hormone production after lipopolysaccharides (LPS) challenge during IVM (*n* = 7 independent experiments). Cumulus–oocyte complexes were cultured for 20–22 h either in control medium alone, or in medium containing 150 ng/mL recombinant human GDF9, in the presence of 0.1 µg/mL *E. coli* LPS, or both 0.1 µg/mL LPS and 150 ng/mL recombinant human GDF9 simultaneously. Estradiol and progesterone concentrations in the spent culture media were determined by radioimmunoassay. A two‐way ANOVA was applied to test for treatment effects and, if the *p* value was < 0.05, a Tukey multiple comparison post hoc test was performed. Concentrations are presented as mean ± SD.

Concentrations of P4 and E2 in the spent IVM media, as well as the E2:P4 ratio, were similar for all groups (*p* > 0.05), regardless of the treatment (Figure 6).

## 4 Discussion

This study aimed to analyze the effects of GDF9 supplementation during IVM on LPS‐treated bovine oocytes as determined by the success of in vitro embryo production, as well as the expression of target genes in cumulus cells and oocytes, and steroid production by cumulus cells. The addition of GDF9 to IVM media containing LPS restored cumulus expansion, cleavage, and blastocyst rates, and the quality of the blastocysts produced was similar to that of the controls. An increase in *CXCL8*, *TNFa*, and *TLR2* transcriptional levels in response to LPS was observed in the cumulus cells, but this effect was absent when GDF9 and LPS were used concomitantly. No changes in the mRNA gene expression were detected for the transcripts associated with the LPS signaling pathway, oocyte meiotic competence, steroidogenesis, apoptosis, or pluripotency, in either cumulus cells or oocytes. Furthermore, E2 and P4 concentrations in the IVM media were similar for all treatments, with no effects detected after GDF9 and/or LPS addition.

Previous studies demonstrated that LPS decreases oocyte competence in vitro and disrupts early embryonic development (Castro et al. 2024; Magata and Shimizu 2017), presumably as a result of the inflammatory milieu produced by the granulosa cells (Tariq et al. 2025). Using a LPS concentration of 0.1 µg/mL during IVM, similar to the mean intrafollicular concentration determined in cows with clinical endometritis (mean 176.1 ng/mL (Herath et al. 2007)), we observed detrimental effects on cumulus expansion, cleavage and blastocyst rates, as well as a significant decrease in embryo quality. Similar effects are observed in vivo, as oocytes from high‐LPS follicles exhibit a greater incidence of morphological abnormalities following IVM (Forrest et al. 2022). Interestingly, developing bovine oocytes are already susceptible to LPS during early follicular development while enclosed in preantral and early antral follicles (Magata et al. 2024; Rahman et al. 2012). Considering that the oocyte acquires its competence when follicles are 2–3 mm in diameter (Lonergan et al. 1994), this might explain the long‐term reproductive dysfunction in cows with postpartum inflammatory disease.

The addition of 175 ng/mL of in‐house produced recombinant mouse GDF9 to bovine oocytes during IVM has been shown to enhance their maturation and development to the blastocyst stage after IVF, although the underlying mechanisms remain unclear (Hussein et al. 2011). Another study demonstrated that supplementation with 200 ng/mL recombinant human GDF9 enhanced the development of bovine embryos (J. Su et al. 2014). We observed a similar effect in our study after using 150 ng/mL of commercially available recombinant human GDF9, a concentration lying within the biologically active window (100–200 ng/mL) (Alam et al. 2018; McNatty et al. 2005), and this confirmation was important before proceeding with the trials, as different effects of GDF9 on granulosa cell function were observed depending on the species of origin (Reader et al. 2016). Addition of GDF9 alone led to the highest rate of cumulus expansion, cleavage, blastocyst, and Code 1 embryos among all groups. Interestingly, when GDF9 and LPS were added simultaneously, the GDF9 was able to counteract the negative effects of LPS and restored these parameters to values similar to those of the controls. One possible way for GDF9 to interact with LPS is through SMAD3, which mediates GDF9 downstream actions after binding to the type I receptor, ALK5 (Mazerbourg et al. 2004), but is also able to inhibit macrophage activation following LPS treatment (Werner et al. 2000). This signaling pathway has been shown to be active in bovine granulosa cells (Spicer et al. 2006). In rodent granulosa cells, inhibition of the SMAD2/3 pathway inhibited cell proliferation induced by the synergistic activity of GDF9 and BMP15 (Reader et al. 2016) and a similar effect was obtained using an NF‐κB inhibitor (Mottershead et al. 2012; Reader et al. 2016). While the NF‐κB signaling pathway is noncanonical for GDF9, it is essential for LPS signaling.

As the mechanisms by which GDF9 interacts with LPS are not understood yet, we decided to analyze the relative mRNA expression of target genes in cumulus cells treated with either GDF9, LPS, or a combination of both. Bovine cumulus cells responded to the LPS challenge with a dramatic increase in *CXCL8* and *TNFa* transcriptional levels, and this effect was largely counteracted when GDF9 was used simultaneously. At the same time, IL6, another known marker of inflammatory response in bovine granulosa cells (Tariq et al. 2025), was not affected in cumulus cells by any treatment, in line with previous observations (Piersanti et al. 2019). Interestingly, no significant changes in *TLR4* or *NF‐κB2* mRNA levels were observed after treatment with LPS or GDF9, but there was a significant increase in *TLR2* levels following LPS treatment compared to control. LPS induces inflammatory processes in bovine granulosa cells, and in ovine luteal endothelial cells, by activating the NF‐κB signaling pathway via TLR2 and TLR4 (Gram and Kowalewski 2022; Price et al. 2013). While *TLR4* has previously been shown to remain unaffected by LPS challenge in bovine cumulus cells (Alvarado Rincón et al. 2019), this is the first study to demonstrate increased *TLR2* and steady *NF‐κB2* mRNA expression in these cells following LPS treatment. Consequently, we hypothesized that LPS might use alternative signaling pathways in cumulus cells, or that it might not exert direct effects on them. Nevertheless, posttranscriptional effects cannot be ruled out, as LPS has been shown to modify protein activity without affecting protein expression in ovine luteal cells (Gram et al. 2019).

In contrast to reports in granulosa cells, the addition of LPS and/or GDF9 did not affect steroid hormone output by cumulus cells, nor the gene expression of *STAR* and *HSD3B*. Bovine cumulus cells are able to secrete E2 and P4 in IVM culture systems, but their concentrations are relatively low in the absence of a substrate (Mingoti et al. 2002), similar to our observations. It should be noted, however, that the COCs were covered by mineral oil during IVM in this study, and this overlay significantly reduces P4, but not E2, concentration in IVM media (Blaschka et al. 2021; Shimada et al. 2002). Previously, GDF9 was shown to reduce E2 and P4 production by bovine granulosa cells by suppressing aromatase activity but only in high dosages (300–600 ng/mL) and only in the presence of IGF1 and FSH (Spicer et al. 2006). However, an effect of GDF9 on the steroidogenic activity of granulosa cells cannot be excluded, based on the dynamic changes in its intrafollicular concentration as well as the negative correlation between *GDF9* and *STAR* expression (Samie et al. 2025). Besides being involved in steroidogenesis, GDF9 is also able to support cell proliferation, while reducing apoptosis in bovine cumulus cells (Fu et al. 2023). Here, we did not observe any effects of either GDF9 or LPS on mRNA availability of the apoptosis‐related genes *BAX* and *BCL2* in cumulus cells. Similar observations related to LPS effects in cumulus cells were made by others using a different set of apoptosis‐related genes (Piersanti et al. 2019). Moreover, that study suggested that LPS may exert stronger apoptotic effects on oocytes than on cumulus cells, which translates into increased apoptosis in the resulting blastocysts during early embryonic development (Tariq et al. 2025).

Having observed the contradictory effects of LPS and GDF9 on cumulus expansion and oocyte developmental competence after IVF, we explored the expression of various genes that were previously associated with either of these two processes. Thymosins 4 and 10 (TMSB4 and TMSB10) are upregulated in bovine cumulus cells during IVM but their expression is not associated with early development in vitro (Salhab et al. 2010; Turhan et al. 2020). Similar to the thymosins, GATM, which encodes a mitochondrial enzyme involved in creatine biosynthesis, is increased in bovine cumulus cells after IVM and also in those COCs that develop into a blastocyst compared to those that arrest at an early stage (Bunel et al. 2015; Turhan et al. 2020). Our analyses of *TMSB4*, *TMSB10*, and *GATM* mRNA expression in cumulus cells after IVM showed no effect of treatment with GDF9 or LPS, most likely because we only assessed cumulus cells after IVM and not before. Cumulus expansion is an essential process for ovulation and it requires the synthesis of hyaluronic acid in large amounts in response to the LH surge (L. Chen et al. 1994). The synthesis is mediated by hyaluronan synthase 2 (HAS2), but it is also supported by epidermal growth factor‐like ligands such as amphiregulin (AREG) (Park et al. 2004). Besides cumulus expansion, AREG is also involved in oocyte maturation and ovulation by mediating LH surge‐regulated ovarian functions in an autocrine and/or paracrine manner (Fang et al. 2023). While GDF9 was shown to have a stimulatory effect on *HAS2* expression in cultured bovine and porcine cumulus cells (Lin et al. 2014; Liu et al. 2018), the opposite was observed in mice following intraperitoneal injection with LPS (Shepel et al. 2018).

In our study, treatment with GDF9 or LPS during IVM did not affect *HAS2* transcriptional levels in bovine cumulus cells and oocytes. Previously, an increase in *HAS2* levels of bovine oocytes was reported following treatment with 10 and 10000 ng/mL LPS during IVM, but not with 100 ng/mL (Piersanti et al. 2019), as used in our study. Furthermore, we could not detect any treatment effects on *AREG* gene expression in cumulus cells and oocytes, confirming previous observations by others (Alvarado Rincón et al. 2019; Piersanti et al. 2019). In comparison, AREG expression and serum concentration are increased in a mouse sepsis model following LPS administration (Yuan et al. 2025).

In our study, *POU5F1* and *SOX2* mRNA expression was detected in bovine oocytes and cumulus cells, without being affected by treatment with LPS. While our observations regarding *POU5F1* expression resemble those of others when using the same LPS dose as here, we could not reproduce the reported increase in *SOX2* mRNA expression in bovine cumulus cells following LPS treatment (Piersanti et al. 2019). This was most likely due to the high variation observed in the LPS group. In comparison to LPS, there is a lack of information regarding the effects of GDF9 on the expression of pluripotency markers in oocytes and cumulus cells. Coculture of porcine oocytes with human endothelial progenitor cells during IVM results in increased *GDF9* expression, which in turn enhances the expression of pluripotency markers such as *POU5F1* and *SOX2* in porcine oocytes and developing blastocysts (Lee et al. 2018). This suggests that GDF9 not only participates in oocyte maturation processes but also maintains pluripotency in early embryonic stages. Although GDF9 supplementation did not lead to any changes in transcriptional levels of pluripotency markers in oocytes and cumulus cells in this study, it cannot be excluded that analysis of blastocysts following IVC might have shown different results.

## 5 Conclusion

This study sheds new light on the involvement of GDF9 and LPS in bovine oocyte maturation and early embryonic development. Our results demonstrate that GDF9 supplementation during IVM of LPS‐treated bovine oocytes rescues their developmental competence without causing major transcriptional changes in the steroidogenic activity of cumulus cells or in the expression of target genes in oocytes and cumulus cells. However, posttranscriptional effects cannot be excluded, and these were not analyzed in this study as proteins were not available. Following up on the developmental rates of the oocytes from which the cumulus cells used for mRNA expression analysis originated may be helpful, as considerable variation in the transcriptional levels of some genes, especially after LPS treatment, was observed. Nevertheless, the positive effects of GDF9 on embryonic development should be confirmed by analyzing the pregnancy rates obtained after transfer of the specific embryos.

## Author Contributions

**Dragos Scarlet:** conceptualization, data curation, investigation, methodology, writing – original draft. **Idil Serbetci:** investigation, methodology, writing – review and editing. **Gerhard Schuler:** investigation, methodology, writing – review and editing. **Heiner Bollwein:** resources, supervision, writing – review and editing. **Mariusz P. Kowalewski:** resources, supervision, data curation, methodology, writing – review and editing.

## Conflicts of Interest

The authors declare no conflicts of interest.

## Data Availability

The data supporting this study's findings are available from the corresponding author upon reasonable request.
